# Goals, Expectations, and the Definition of Success for Neuromodulation for Pain According to Representatives of Neuromodulation Device Manufacturers

**DOI:** 10.3390/jpm12091457

**Published:** 2022-09-06

**Authors:** Maarten Moens, William Alliet, Maxime Billot, Ann De Smedt, Panagiotis Flamée, Domien Vanhonacker, Manuel Roulaud, Philippe Rigoard, Lisa Goudman

**Affiliations:** 1 Department of Neurosurgery, Universitair Ziekenhuis Brussel, Laarbeeklaan 101, 1090 Brussels, Belgium; 2 Department of Radiology, Universitair Ziekenhuis Brussel, Laarbeeklaan 101, 1090 Brussels, Belgium; 3 STIMULUS Consortium (reSearch and TeachIng neuroModULation Uz bruSsel), Vrije Universiteit Brussel, Laarbeeklaan 103, 1090 Brussels, Belgium; 4 Center for Neurosciences (C4N), Vrije Universiteit Brussel, Laarbeeklaan 103, 1090 Brussels, Belgium; 5 Pain in Motion (PAIN) Research Group, Department of Physiotherapy, Human Physiology and Anatomy, Faculty of Physical Education and Physiotherapy, Vrije Universiteit Brussel, Laarbeeklaan 103, 1090 Brussels, Belgium; 6 Department of Anesthesiology, Universitair Ziekenhuis Brussel, Laarbeeklaan 101, 1090 Brussels, Belgium; 7 PRISMATICS Lab (Predictive Research in Spine/Neuromodulation Management and Thoracic Innovation/Cardiac Surgery), Poitiers University Hospital, 86021 Poitiers, France; 8 Department of Physical Medicine and Rehabilitation, Universitair Ziekenhuis Brussel, Laarbeeklaan 101, 1090 Brussels, Belgium; 9 Department of Spine Surgery & Neuromodulation, Poitiers University Hospital, 86021 Poitiers, France; 10 Pprime Institute UPR 3346, CNRS, ISAE-ENSMA, University of Poitiers, 86360 Chasseneuil-du-Poitou, France; 11 Research Foundation—Flanders (FWO), 1090 Brussels, Belgium

**Keywords:** goalsetting, industry, neuromodulation, spinal cord stimulation, chronic pain, clinical success, survey

## Abstract

Representatives of neuromodulation device manufacturers are expected to facilitate the relationship between patients and healthcare providers. Nevertheless, the goals, expectations, and definition of success for neuromodulation for pain have not yet been explored. Representatives present at the 2nd Joint Congress of the INS European Chapters in September 2021 completed a survey to ascertain their opinions about the goals to achieve with neuromodulation, the factors that they expect to change, and their definition of success for neuromodulation. In total, 39 representatives completed the survey. To provide excellent service for patients (22.4%), to become a trusted partner for physicians (21.5%), and to provide excellent service for physicians (20.7%) were the highest ranked goals. The most frequently reported factors that were expected to change were pain intensity (23.1%), patient satisfaction (19.7%), mobility/functioning (14.5%), and capacity to return to work (13.7%). Within the definitions of success, increased quality of life of the patient was stated in 21% of the definitions, closely followed by pain control (19.3%) and happiness/patient satisfaction (15.8%). The goals of representatives of neuromodulation device manufacturers seem to focus on ensuring a good relationship with physicians on the one hand and providing good service towards patients on the other hand, whereby pain control, quality of life, and patient satisfaction seem to be important for company representatives.

## 1. Introduction

Chronic pain management and associated neuromodulation strategies are domains where decades of clinical and research experience have been conducted, with ongoing developments in terms of implantation strategy [1], patient selection [2,3], and the underlying mechanism of action [4]. Treating patients with chronic pain often proves challenging, whereby the most optimal result is obtained when an interdisciplinary team approach is applied [5]. In addition to the patient and their personal environment, the healthcare environment and, commonly related to the field of neuromodulation, representatives of neuromodulation device manufacturers are jointly responsible for the management of chronic pain and striving towards the most optimal result.

Firstly, patients should be individually treated following a biopsychosocial multidisciplinary pain management approach, incorporating the perceptions, cognitions, and emotions of the patient, in addition to the potential biomedical dysfunction [6,7,8,9]. Additionally, relevant treatment goals should be one of the cornerstones of a successful rehabilitation scheme for chronic pain patients [10], whereby a European survey denoted that taking part in family and social activities, pain reduction, and household tasks were the highest ranked goals for patients with chronic pain [11].

Secondly, the full team of healthcare providers should strive toward a tailored-made treatment plan for each patient with chronic pain [12]. Especially in relation to neuromodulation, the implanting physicians should weigh the reductions in patient-reported symptoms and increased functionality and health-related quality of life against the risks for de-vice-related complications, infections, or adverse events of the stimulation [13]. Healthcare providers should create an environment in which patients can maximally benefit from the device, by aligning expectations concerning the stimulation, creating realistic goals, and correctly informing the patient and family members concerning short-term and long-term therapy effectiveness [13,14]. Hence, the relation between patients and the healthcare team cannot be ignored [15], whereby a shared decision-making process is an essential prerequisite to avoid different perceptions of both stakeholders (i.e., patient (and his/her environment) and healthcare providers) [16].

Specifically for neuromodulation, a third important stakeholder is the industry responsible for manufacturing, promoting, and programming the neuromodulation devices. Representatives of neuromodulation device manufacturers have an in-depth understanding of the nuances of each neuromodulation product, which is highly beneficial to select the most appropriate device for each individual patient [17]. Previous research implied that the knowledge, the information transfer, the communication skills, and professionalism of the medical representatives influence the physicians’ habitual behavior towards the prescription of drugs [18]. Similarly, medical promotional tools provided by medical representatives have an influence on physicians’ prescribing practices [19]. Research on the exact influence of medical representatives in the field of neuromodulation is still limited; however, it might be assumed that representatives of neuromodulation device manufacturers influence both patients and physicians when considering the process of initiating a trajectory with neuromodulation. This study aimed to gain insight into the beliefs of representatives of neuromodulation device manufacturers concerning the success of neuromodulation. Therefore, the goals of this study were to gain insight into the opinion of representatives of neuromodulation device manufacturers on three aspects, namely (i) the goals of neuromodulation, (ii) the factors that are expected to change due to neuromodulation for pain, and (iii) the definition of success for neuromodulation for pain.

## 2. Materials and Methods

### 2.1. Participants

During the 2nd Joint Congress of the INS European Chapters in September 2021 in Paris, representatives of neuromodulation device manufacturers were asked to complete an online survey. This congress was held in accordance with the ongoing COVID-19 regulations. In this cross-sectional online survey, all male and female representatives of neuromodulation device manufacturers with a decent knowledge of English could participate, since all questions were provided in English. The sampling frame was all representatives of neuromodulation device manufacturers who were physically able to participate at this congress. The authors asked all industry representatives to complete the survey by personally explaining the study to each representative separately and asking them to scan the QR code to complete the survey. The survey was filled in anonymously, without a financial or other incentive for survey respondents. Prior to initiating this study, the survey was registered at ClinicalTrials.gov, accessed on 2 September 2022 with number NCT05013827. The study protocol was approved by the central ethics committee of Universitair Ziekenhuis Brussel (B.U.N. 1432021000550) on 28 July 2021.

### 2.2. Data Collection

A multidisciplinary team of healthcare providers, including pain physicians (neurosurgery), physiotherapists, epidemiologists, and biostatisticians developed the survey questions. The final survey was constructed to be completed within 10 min. Before finalizing the survey, the technical functionality and logical order of the survey were pilot tested by two persons with different backgrounds. The survey was available from 2 September 2021 to 4 September 2021 at the 2nd Joint Congress of the INS European Chapters (e-INS 2021) in Paris. In order to guarantee that the survey was anonymous, IP addresses were not saved, and respondents were not asked to provide the name of the company they were working for.

The online survey consisted of five different questions, including two general questions to explore the experience that respondents had within their company and to ask their profession. The goals for neuromodulation for pain and factors that respondents expected to change according to neuromodulation for pain were evaluated with a predefined list of answer options. The goals were defined as the intended future state after a certain intervention [20], i.e., neuromodulation for pain in this case. Respondents were requested to select their top three answers from the list or to add another item if their choice was not mentioned in the list. As a final question, respondents were asked to provide their definition of success for the neuromodulation for pain (open question) to gain insight into what the representatives of neuromodulation device manufacturers considered a response with respect to this therapy. The exact questions from the survey are presented in Supplementary Material S1.

### 2.3. Data Analysis

All data were collected through Qualtrics. Analyses were performed in R studio version 1.4.1106 (R version 4.0.5). All analyses were based on the actual response data, meaning that there were no imputation strategies employed. Descriptive statistics are provided as mean (±standard deviation), median (first and third quartile), or number of observations (percentage). For goals that were specified by the respondents themselves, content analysis was performed. Results are presented for all representatives of neuromodulation device manufacturers together, as well as separately for representatives with direct patient contact and representatives belonging to higher management.

## 3. Results

### 3.1. Survey Respondents

The survey was launched at e-INS on 2 September 2021. All representatives of the neuromodulation device manufacturers who were present at the congress at the industry booth (2–4 September 2021) were personally invited by the authors to complete the survey and spread it throughout their company. According to the congress administration, 16% of attendees were industry/corporate professionals, leading to an estimated presence of 232 representatives who registered. At the congress, representatives of the following compagnies were present: Abbott, Bioness, Boston Scientific, Mainstay Medical, Medtronic, Neurimpulse, Nevro, Saluda Medical, and Stimwave. The survey was still accessible during the weeks after the congress. In total, 46 respondents opened the link to the survey, and 39 representatives eventually completed the survey. The first response was collected on 3 September 2021, and the last completed response was received on 9 September 2021.

The medium time to complete the survey was 222 s (Q1–Q3: 150–410). Representatives with different profiles within their company completed the survey, namely four respondents had a clinical profession within their company, fifteen respondents were higher management representatives, thirteen were sales representatives (patient contact), four were marketing representatives, two were engineers, and one respondent did not specify the exact profession. Most of the respondents had long-term experience with neuromodulation, i.e., fourteen respondents had between 10 and 20 years of experience, and two respondents had more than 20 years of experience. Eleven respondents had 5–10 years of experience, seven respondents had 3–5 years of experience, four respondents had 1–3 years of experience, and one person had less than 1 year of experience within this specific field.

### 3.2. Goals and Influenceable Factors

One respondent only selected two goals, leading to a total of 116 goals that were mentioned. The top three goals that were selected are presented in Table 1.

**Figure 1:**
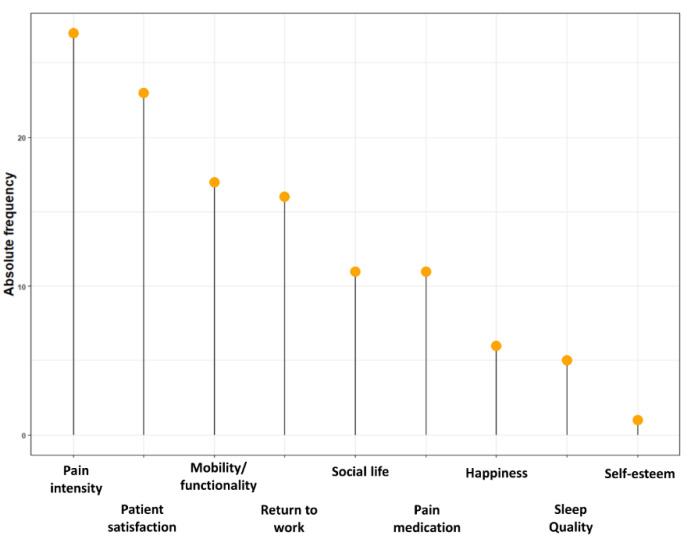
Plot representing the top three factors that representatives of neuromodulation device manufacturers believe will change due to neuromodulation for pain. On the y-axis, the absolute frequency, i.e., the total number of responses, is presented.

The goals that were most often selected were providing excellent service at the highest standards for patients (22.4%), to become a trusted partner for physicians (21.5%), and providing excellent service at the highest standards for physicians (20.7%). The most frequently reported factors that were expected to change due to neuromodulation, according to representatives of neuromodulation device manufacturers, were pain intensity (23.1%), patient satisfaction (19.7%), mobility/functioning (14.5%), and the capacity to return to work (13.7%) (Figure 1). Separate results for representatives with patient contact and higher management representatives are presented in Table 2.

### 3.3. Success of Neuromodulation

Representatives of neuromodulation device manufacturers were also asked to provide the definition of success for neuromodulation for pain, according to their point of view (Table 3).

**Figure 2:**
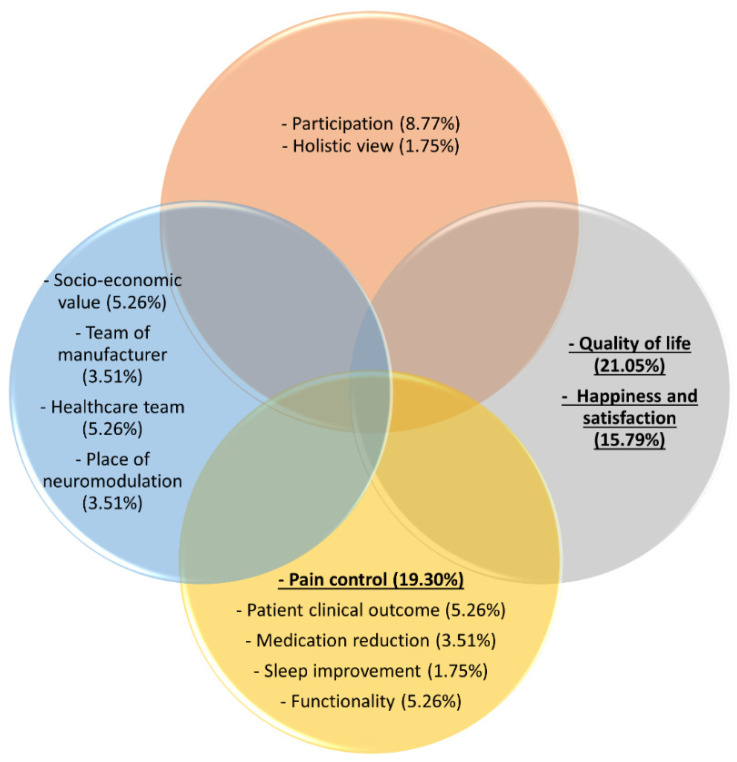
Extracted components from the definition of success for neuromodulation for pain according to representatives of neuromodulation device manufacturers. The yellow circle represents bodily functions and structures, the orange circle represent participation, the blue circle represent industry-related aspects, and the grey circle represent health-related quality of life. The three most frequently stated components are underlined and presented in bold.

Of the 39 representatives who completed the survey, four did not provided an answer to this question; hence, 35 definitions were recorded. These 35 definitions led to the identification of 57 components that were extracted from these definitions (Figure 2).

The increased quality of life of the patient was stated 12 times (21%), closely followed by pain control, which was mentioned 11 times (19.3%). A third component that was frequently identified was happiness, including patient satisfaction (N = 9, 15.8%). Patient participation (i.e., return to work, participation in social life, and achieving predetermined goals) was mentioned five times (8.8%) as an important component of the definition of success of neuromodulation for pain. Functionality, meaning improvements in mobility/functionality and increase in activities, was selected three times (5.3%), a decrease in medication use twice (3.5%), and sleep improvement once (1.8%). Additionally, overall patient clinical outcome improvements without a specific definition were stated three times (5.3%). The socioeconomic value, which entails the profit and loss outcome, satisfied customers, and the long-term reimbursement criteria and solutions, was stated three times (5.3%). The place of neuromodulation in the treatment options was stated twice (3.5%). The team support was mentioned twice in relation to the team of the manufacturer (3.5%). Furthermore, the team support in relation to the team of healthcare providers was mentioned three times, whereby less stress for healthcare providers, satisfied physicians, and the independence of the industry’s support as a healthcare team were specifically mentioned (5.3%). Finally, a holistic vision to define success with looking further than only suppressing pain was mentioned once (1.8%).

## 4. Discussion

Most fields in medicine involve a close cooperation of industry/medical representatives during daily clinical practice, among which are neuromodulation or cardiology for implantable devices and prescribing physicians for pharmaceutical products. With this involvement, representatives are capable of influencing physicians [21], for example, by providing prescribers pens, notepads, and other tools to ensure that a targeted drug’s name or company name stays subconsciously in the mind of the prescribers [22]. Nevertheless, in the field of neuromodulation, the point of view of industry representatives has not yet been explored, and it is not clear whether they have a different point of view on the effect of neuromodulation for pain compared to healthcare providers. This study explored the goals and factors that representatives of neuromodulation device manufacturers expect to change according to neuromodulation for pain. Providing excellent service at the highest standards for patients (22.4%), to become a trusted partner for physicians (21.5%), and providing excellent service at the highest standards for physicians (20.7%) were the highest ranked goals to achieve with neuromodulation for pain. These goals clearly denote that representatives of neuromodulation device manufacturers aim to provide excellent service for both the team of healthcare providers as well as for patients themselves, where representatives of neuromodulation device manufacturers could play a mediating role between both stakeholders. An interesting finding is that when accumulating these goals, 22.4% of the respondents aimed to provide excellent service for patients, while 42.2% aimed to become a trusted partner for physicians or provide excellent service for physicians. Hypothetically, this could be explained by the continuing relationship that company representatives must maintain with physicians and to a lesser extent with individual patients. Remarkably, health-economic aspects, economic value, and innovations were less frequently selected as goals, although these aspects are inevitably related to industrial partners. However, when specifically evaluating the goals of representatives belonging to higher management, it becomes clear that an impact on health-economic aspects was more important to them, as compared to representatives with patient contact, since 17.8% versus 5.3% selected this as a goal, respectively. To become a key leader in innovation was not selected by representatives from higher management, which was a result that was not expected.

The most frequently reported factors that were expected to change due to neuromodulation were pain intensity (23.1%), patient satisfaction (19.7%), mobility/functioning (14.5%), and the capacity to return to work (13.7%). During this congress, healthcare providers were also questioned about the same topics, whereby pain intensity (27.2%), followed by mobility/functionality (26%) and pain medication use (18.7%), were denoted as factors that are expected to change due to neuromodulation for pain [23]. As such, the main expectations of representatives of neuromodulation device manufacturers and healthcare providers are in line with each other concerning pain intensity and mobility/functionality. Apparently, representatives of neuromodulation device manufacturers expect an influence on the capacity to return to work, which is not an aspect that is supported by healthcare providers. A meta-analysis previously denoted that SCS results in a higher prevalence of patients at work compared with the situation before SCS (OR 2.15, 95% CI 1.44–3.21) [24]; however, the percentages of patients that effectively resumed work after SCS ranged between 10% [25] and 47% [26]. On the contrary, healthcare providers were more prone to select a reduction in pain medication use as a factor that is influenced by SCS, a finding that is completely in line with recent findings in the literature [27,28].

Within the definitions of success of neuromodulation, increased quality of life of the patient was stated in 21% of the definitions, closely followed by pain relief (19.3%) and happiness, including patient satisfaction (15.8%). Representatives seem to denote quality of life as an important contributor to the definition of success of neuromodulation, an aspect that has gained a lot of attention lately with respect to the creation of composite endpoints to evaluate treatment effects in neuromodulation [29,30,31,32]. Additionally, generic health-related quality of life questionnaires, including the EQ5D or SF-36 [33], are the basis for calculating quality-adjusted life years (a measure used to quantify health outcomes) in cost–utility analysis to guide health care resource allocation [34]. Surprisingly, functionality (including mobility) was only included in the definition of success in 5.3% of the responses. Nevertheless, this factor belonged to the top three factors that were expected to change due to neuromodulation for pain. Apparently, the increase in functionality that is expected to occur after neuromodulation is not a criterion as such to determine whether neuromodulation is successful or not. It may be suggested that the translation of an increased functionality towards a higher quality of life or regaining a feeling of happiness and patient satisfaction is the criterion to classify the treatment as successful and not the increased functionality itself if this does not result in a higher goal. This hypothesis would be in line with the results of the European survey in chronic pain patients, in which goals were situated at the household level and family/social activities and not at an increased functionality level [11]. Finally, only respondents from higher management included components of the collaboration within the manufacturers team and the cooperation with the healthcare team in the definition of success of neuromodulation. Representatives with direct patient contact did not include these components, but rather included patient reported outcomes among which sleep improvement and a reduction in medication use were included in their definitions of success. These elements may suggest that the position of representatives influences their judgement to whether a patient is responding to the treatment or not; however, further research is needed to confirm this statement.

This survey was able to provide useful insights into the goals and definitions of success that industrial partners of neuromodulation devices consider important. Nevertheless, the presence of selection bias could not be excluded with this type of study design. Additionally, the limited number of respondents was a shortcoming of this survey. Finally, this survey was launched at the 2nd Joint Congress of the INS European Chapters, which was mainly attended by European company representatives. Therefore, this survey mainly represents the opinion of European company representatives and may not be generalizable to the opinion of company representatives outside Europe. Similarly, this survey may not be representative for the true mission and vision of neuromodulation device companies but presents the view of company representatives in relation to neuromodulation. Despite the disputable reputation of industry funders in relation to performance bias, positive results, and reporting of randomized controlled trials in medicine [35,36,37,38], this survey suggested that patients, healthcare providers, and representatives of neuromodulation device manufacturers mainly have comparable ideas concerning the goals for neuromodulation. This suggests that the influence that company representatives may induce on the therapeutic relation between patients and healthcare providers will be in a similar direction.

## 5. Conclusions

This survey demonstrated that company representatives of neuromodulation devices for pain attached importance to a good relationship with physicians as well as good service for patients. Pain control, quality of life, and patient satisfaction seem to be important factors for successful therapy according to company representatives.

## Figures and Tables

**Table 1:** Survey respondents reported a total of 116 goals for neuromodulation for pain. The absolute frequency and percentage of the top three goals that were selected by representatives of neuromodulation device manufacturers are presented. Percentages are calculated according to the number of goals that were selected for each group, meaning 116 goals for all respondents, 38 goals for respondent with patient contact, and 45 goals for respondents belonging to higher management.

Goals of Representatives of Neuromodulation Device Manufacturers	All Respondents (N = 39)	Respondents with Patient Contact (N = 13)	Respondents from Higher Management (N = 15)
To improve the economic value of your company	9 (7.8%)	3 (7.9%)	4 (8.9%)
To have a major impact on health-economic aspects	12 (10.3%)	2 (5.3%)	8 (17.8%)
To become a key opinion leader in innovation	8 (6.9%)	3 (7.9%)	0 (0.0%)
To become a trusted partner for physicians	25 (21.5%)	9 (23.7%)	8 (17.8%)
To provide excellent service at the highest standards for physicians	24 (20.7%)	9 (23.7%)	9 (20.0%)
To provide excellent service at the highest standards for patients	26 (22.4%)	8 (21.0%)	11 (24.4%)
To improve my personal technical/marketing/management skills	3 (2.6%)	2 (5.3%)	0 (0.0%)
To have superior quality/superior clinical results/etc. compared to our competitors	7 (6.0%)	2 (5.3%)	3 (6.7%)
To have the highest impact on national regulations	1 (0.9%)	0 (0.0%)	1 (2.2%)
To have the broadest portfolio in neuromodulation for pain	1 (0.9%)	0 (0.0%)	1 (2.2%)

**Table 2:** Survey respondents reported a total of 117 factors that are expected to change with neuromodulation for pain. The absolute frequency and percentage of the top three factors that were selected by representatives of neuromodulation device manufacturers are presented. Percentages are calculated according to the number of factors selected for each group, meaning 117 factors for all respondents, 39 factors for respondent with patient contact, and 45 factors for respondents belonging to higher management.

Expectations of Representatives of Neuromodulation Device Manufacturers	All Respondents (N = 39)	Respondents with Patient Contact (N = 13)	Respondents from Higher Management (N = 15)
Pain intensity	27 (23.1%)	8 (20.5%)	9 (20.2%)
Mobility/functionality	17 (14.5%)	4 (10.2%)	6 (13.3%)
Pain medication use	11 (9.4%)	4 (10.2%)	5 (11.1%)
Sleep quality	5 (4.3%)	3 (7.7%)	0 (0.0%)
Capacity to return to work	16 (13.7%)	6 (15.4%)	9 (20.2%)
Participation in social life	11 (9.4%)	4 (10.2%)	5 (11.1%)
Self-esteem	1 (0.8%)	0 (0.0%)	0 (0.0%)
Feeling of happiness	6 (5.1%)	4 (10.2%)	1 (2.2%)
Patient satisfaction	23 (19.7%)	6 (15.4%)	10 (22.2%)

**Table 3:** Extracted components from the definition of success for neuromodulation for pain according to representatives of neuromodulation device manufacturers. Separate results are provided for representatives with direct patient contact and representatives from higher management.

Components Included in Definition of Success of Neuromodulation for Pain	All Respondents (N = 35)	Respondents with Patient Contact (N = 13)	Respondents from Higher Management (N = 14)
Pain control	11 (19.3%)	5 (22.7%)	3 (15.8%)
Quality of life	12 (21%)	4 (18.2%)	5 (26.3%)
Happiness and satisfaction	9 (15.8%)	2 (9.1%)	1 (5.3%)
Participation	5 (8.8%)	1 (4.5%)	3 (15.8%)
Holistic view	1 (1.8%)	1 (4.5%)	0 (0.0%)
Socioeconomic value	3 (5.3%)	2 (9.1%)	1 (5.3%)
Team of manufacturer	2 (3.5%)	0 (0.0%)	2 (10.5%)
Healthcare team	3 (5.3%)	0 (0.0%)	1 (5.3%)
Place of neuromodulation	2 (3.5%)	1 (4.5%)	1 (5.3%)
Patient clinical outcome	3 (5.3%)	2 (9.1%)	1 (5.3%)
Medication reduction	2 (3.5%)	2 (9.1%)	0 (0.0%)
Sleep improvement	1 (1.8%)	1 (4.5%)	0 (0.0%)
Functionality	3 (5.3%)	1 (4.5%)	1 (5.3%)

## Data Availability

Data available on motivated request of the author.

## Supplementary Materials

The following supporting information can be downloaded at: https://www.mdpi.com/article/10.3390/jpm12091457/s1.

## References

[1] Wood C., Martiné G., Espagne-Dubreuilh G., Le Goff K., Moens M., Goudman L., Baron S., David R., Naïditch N., Billot M. (2022). The Added Value of Intraoperative Hypnosis during Spinal Cord Stimulation Lead Implantation under Awake Anesthesia in Patients Presenting with Refractory Chronic Pain. Medicina.

[2] Thomson S., Huygen F., Prangnell S., De Andrés J., Baranidharan G., Belaïd H., Berry N., Billet B., Cooil J., De Carolis G. (2020). Appropriate referral and selection of patients with chronic pain for spinal cord stimulation: European consensus recommendations and e-health tool. Eur. J. Pain.

[3] Goudman L., Rigoard P., Billot M., Duarte R.V., Eldabe S., Moens M. (2022). Patient selection for Spinal Cord Stimulation in treatment of pain: A sequential decision-making model. A narrative review. J. Pain Res..

[4] Goudman L., De Groote S., Linderoth B., De Smedt A., Eldabe S., Duarte R., Moens M. (2021). Exploration of the Supraspinal Hypotheses about Spinal Cord Stimulation and Dorsal Root Ganglion Stimulation: A Systematic Review. J. Clin. Med..

[5] Rigoard P., Gatzinsky K., Deneuville J.-P., Duyvendak W., Naiditch N., Van Buyten J.-P., Eldabe S. (2019). Optimizing the Management and Outcomes of Failed Back Surgery Syndrome: A Consensus Statement on Definition and Outlines for Patient Assessment. Pain Res. Manag..

[6] Malfliet A., Coppieters I., Van Wilgen P., Kregel J., De Pauw R., Dolphens M., Ickmans K. (2017). Brain changes associated with cognitive and emotional factors in chronic pain: A systematic review. Eur. J. Pain.

[7] Wijma A.J., Van Wilgen C.P., Meeus M., Nijs J. (2016). Clinical biopsychosocial physiotherapy assessment of patients with chronic pain: The first step in pain neuroscience education. Physiother. Theory Pract..

[8] Naiditch N., Billot M., Moens M., Goudman L., Cornet P., Le Breton D., Roulaud M., Ounajim A., Page P., Lorgeoux B. (2021). Persistent Spinal Pain Syndrome Type 2 (PSPS-T2), a Social Pain? Advocacy for a Social Gradient of Health Approach to Chronic Pain. J. Clin. Med..

[9] Naiditch N., Billot M., Goudman L., Cornet P., Roulaud M., Ounajim A., Page P., Lorgeoux B., Baron S., Nivole K. (2021). Professional Status of Persistent Spinal Pain Syndrome Patients after Spinal Surgery (PSPS-T2): What Really Matters? A Prospective Study Introducing the Concept of “Adapted Professional Activity” Inferred from Clinical, Psychological and Social Influence. J. Clin. Med..

[10] Dekker J., De Groot V., Ter Steeg A.M., Vloothuis J., Holla J., Collette E., Satink T., Post L., Doodeman S., Littooij E. (2019). Setting meaningful goals in rehabilitation: Rationale and practical tool. Clin. Rehabil..

[11] Goudman L., De Smedt A., Linderoth B., Eldabe S., Witkam R., Henssen D., Moens M. (2021). Identifying goals in patients with chronic pain: A European survey. Eur. J. Pain.

[12] Chadwick A., Frazier A., Khan T.W., Young E. (2021). Understanding the Psychological, Physiological, and Genetic Factors Affecting Precision Pain Medicine: A Narrative Review. J. Pain Res..

[13] Ba N.A.S., Rosenow J.M. (2021). Ethical Considerations in the Implantation of Neuromodulatory Devices. Neuromodulation Technol. Neural Interface.

[14] Goudman L., Bruzzo A., Van De Sande J., Moens M. (2019). Goal Identification Before Spinal Cord Stimulation: A Qualitative Exploration in Potential Candidates. Pain Pract..

[15] Náfrádi L., Kostova Z., Nakamoto K., Schulz P.J. (2018). The doctor–patient relationship and patient resilience in chronic pain: A qualitative approach to patients’ perspectives. Chronic Illn..

[16] Lambing A., Nichols C.D., Munn J.E., Anderson T.L., Tortella B.J., Witkop M.L. (2017). Patient, caregiver, and provider perceptions of pain and pain management in adolescents and young adults with bleeding disorders. Haemophilia.

[17] Clingan J.A., Patel A., Maher D.P. (2020). Survey of Spinal Cord Stimulation Hardware Currently Available for the Treatment of Chronic Pain in the United States. Front. Pain Res..

[18] Bala K., Sharma K. (2020). Role of Medical Representatives in Influencing Medicine Prescription Behaviour of Doctors. J. Bus. Thought.

[19] Ali K.E., Naser A.Y., Al-Rousan R., Alwafi H., AbuAlhommos A.K., Alsairafi Z.K., Salawati E.M., Samannodi M., Dairi M.S. (2022). The attitude and acceptability towards medical promotional tools and their influence on physicians’ prescribing practices in Jordan and Iraq: A cross-sectional study. BMC Health Serv. Res..

[20] Wade D. (2009). Goal setting in rehabilitation: An overview of what, why and how. Clin. Rehabil..

[21] Workneh B.D., Gebrehiwot M.G., Bayo T.A., Gidey M.T., Belay Y.B., Tesfaye D.M., Kassa T.T. (2016). Influence of Medical Representatives on Prescribing Practices in Mekelle, Northern Ethiopia. PLoS ONE.

[22] Fugh-Berman A., Ahari S. (2007). Following the Script: How Drug Reps Make Friends and Influence Doctors. PLoS Med..

[23] Goudman L., De Smedt A., Billot M., Roulaud M., Rigoard P., Moens M. (2022). Opinions of healthcare providers about neuromodulation for pain: Results of an online survey at the 2nd Joint Congress of the INS European Chapters. Neuromodulation Technol. Neural Interface.

[24] Moens M., Goudman L., Brouns R., Msc A.V.E., De Jaeger M., Huysmans E., Putman K., Verlooy J., Moens M., Msc L.G. (2018). Return to Work of Patients Treated with Spinal Cord Stimulation for Chronic Pain: A Systematic Review and Meta-Analysis. Neuromodulation Technol. Neural Interface.

[25] Szmuda T., Słoniewski P., Ali S., Aleksandrowicz K. (2020). Does Spinal Cord Stimulation Due to Failed Back Surgery Syndrome Lead to Permanent Occupational Disability?. Neuromodulation Technol. Neural Interface.

[26] Gopal H., Fitzgerald J., McCrory C. (2016). Spinal cord stimulation for FBSS and CRPS: A review of 80 cases with on-table trial of stimulation. J. Back Musculoskelet. Rehabil..

[27] Goudman L., De Smedt A., Forget P., Eldabe S., Moens M. (2021). High-Dose Spinal Cord Stimulation Reduces Long-Term Pain Medication Use in Patients with Failed Back Surgery Syndrome Who Obtained at Least 50% Pain Intensity and Medication Reduction During a Trial Period: A Registry-Based Cohort Study. Neuromodulation Technol. Neural Interface.

[28] Pollard E.M., Lamer T.J., Moeschler S.M., Gazelka H.M., Hooten W.M., Bendel M.A., Warner N.S., Murad M.H. (2019). The effect of spinal cord stimulation on pain medication reduction in intractable spine and limb pain: A systematic review of randomized controlled trials and meta-analysis. J. Pain Res..

[29] Pilitsis J.G., Fahey M., Custozzo A., Chakravarthy K., Capobianco R. (2020). Composite Score Is a Better Reflection of Patient Response to Chronic Pain Therapy Compared with Pain Intensity Alone. Neuromodulation Technol. Neural Interface.

[30] Goudman L., De Smedt A., Eldabe S., Rigoard P., Linderoth B., De Jaeger M., Moens M., Consortium D. (2020). High-dose spinal cord stimulation for patients with failed back surgery syndrome: A multicenter effectiveness and prediction study. Pain.

[31] Goudman L., Billot M., Duarte R.V., Eldabe S., Rigoard P., Moens M. (2021). Gradation of Clinical Holistic Response as New Composite Outcome to Evaluate Success in Spinal Cord Stimulation Studies for Pain. Neuromodulation Technol. Neural Interface.

[32] Rigoard P., Ounajim A., Goudman L., Louis P.-Y., Yousri S., Roulaud M., Bouche B., Wood C., Page P., Lorgeoux B. (2021). A Novel Multi-Dimensional Clinical Response Index Dedicated to Improve Pain Global Assessment in Patients with Persistent Spinal Pain Syndrome after Spinal Surgery, Based on a Real-Life Prospective Multicentric Study (PREDIBACK) and Machine Learning Techniques. J. Clin. Med..

[33] Kaplan R.M., Hays R.D. (2022). Health-Related Quality of Life Measurement in Public Health. Annu. Rev. Public Health.

[34] Yang F., Devlin N., Luo N. (2018). Cost-Utility Analysis Using EQ-5D-5L Data: Does How the Utilities Are Derived Matter?. Value Health.

[35] Liang F., Zhu J., Mo M., Zhou C., Jia H., Xie L., Zheng Y., Zhang S. (2018). Role of industry funders in oncology RCTs published in high-impact journals and its association with trial conclusions and time to publication. Ann. Oncol..

[36] Wareham K.J., Hyde R.M., Grindlay D., Brennan M.L., Dean R.S. (2017). Sponsorship bias and quality of randomised controlled trials in veterinary medicine. BMC Veter Res..

[37] Flacco M.E., Manzoli L., Boccia S., Capasso L., Aleksovska K., Rosso A., Scaioli G., De Vito C., Siliquini R., Villari P. (2015). Head-to-head randomized trials are mostly industry sponsored and almost always favor the industry sponsor. J. Clin. Epidemiol..

[38] Bicket M.C., Dunn R.Y., Ahmed S.U. (2016). High-Frequency Spinal Cord Stimulation for Chronic Pain: Pre-Clinical Overview and Systematic Review of Controlled Trials. Pain Med..

