# Supplementary material for: Goals, Expectations, and the Definition of Success for Neuromodulation for Pain According to Representatives of Neuromodulation Device Manufacturers
Source: J Pers Med. 2022 Sep 6;12(9):1457. doi: 10.3390/jpm12091457 (PMC9500654; doi:10.3390/jpm12091457)
Supplement: Supplementary file 1 [file jpm-12-01457-s001.zip › jpm-1881117-supplementary.pdf]

Supplementary Material SI: Survey Questions

How much experience do you have in your company?

- 0 – 1 year
- 1 – 3 years
- 3 – 5 years
- 5- 10 years
- 10 – 20 years
- More than 20 years of experience

What is your profession?

- Sales
- Clinical
- Higher management
- Other, please specify your profession: …………….

What are your **goals** in treating patients with neuromodulation for pain?
Please specify your top 3 goals.

- To improve the economical values of your company
- To have a major impact on health-economical aspects
- To become a key opinion leader in innovation
- To become a trusted partner for physicians
- To provide excellent service at the highest standards for physicians
- To provide excellent service at the highest standards for patients
- To improve my personal technical / marketing / management skills
- To have superior quality / superior clinical results / etc. compared to our competitors
- To have the highest impact on national regulations
- To have the broadest portfolio in neuromodulation for pain
- Other goal, please specify: ……………………………….

What factors do you **expect** to change with neuromodulation for pain?
Please specify your top 3 goals.

- Pain intensity
- Mobility / functionality
- Pain medication use
- Sleep quality
- Capacity to return to work
- Participation in social life
- Self-esteem

- Feeling of happiness
- Patient satisfaction
- Other factors, please specify: ………………………………..

How do you **define success** of neuromodulation for pain?

…….

We thank you for your time spent taking this survey.

Your response has been recorded.
